# Novel circulating peptide biomarkers for esophageal squamous cell carcinoma revealed by a magnetic bead-based MALDI-TOFMS assay

**DOI:** 10.18632/oncotarget.8123

**Published:** 2016-03-16

**Authors:** Kun Jia, Wei Li, Feng Wang, Haixia Qu, Yuanyuan Qiao, Lanping Zhou, Yulin Sun, Qingwei Ma, Xiaohang Zhao

**Affiliations:** ^1^ State Key Laboratory of Molecular Oncology, Cancer Hospital, Chinese Academy of Medical Sciences and Peking Union Medical College, Beijing 100021, China; ^2^ Bioyong Technologies Inc., Beijing 100080, China; ^3^ Center for Basic Medical Sciences, Navy General Hospital, Beijing 100048, China

**Keywords:** circulating peptide markers, ESCC, TSP1, AHSG, FGA

## Abstract

Esophageal squamous cell carcinoma (ESCC) is one of the most common malignant neoplasms worldwide. Patients are often diagnosed at advanced stages with poor prognosis due to the absence of obvious early symptoms. Here, we applied a high-throughput serum peptidome analysis to identify circulating peptide markers of ESCC. Weak cationic exchange magnetic beads coupled to matrix-assisted laser desorption/ionization time-of-flight mass spectrometry was used for two-stage proteotypic peptide profiling in complex serum samples collected from 477 cancer patients and healthy controls. We established a genetic algorithm model containing three significantly differentially expressed peptides at 1,925.5, 2,950.6 and 5,900.0 Da with a sensitivity and specificity of 97.00% and 95.92% in the training set and 97.03% and 100.00% in the validation set, respectively. The model's diagnostic capability was significantly better than SCC-Ag and Cyfra 21–1, especially for early stage ESCC, with an achieved sensitivity of 96.94%. Subsequently, these peptides were identified as fragments of AHSG, TSP1 and FGA by linear ion trap-orbitrap hybrid tandem mass spectrometry. Notably, increased tissue and serum levels of TSP1 in ESCC were verified and correlated with disease progression. In addition, tissue TSP1 was an independent poor prognostic factor in ESCC. In conclusion, the newly established circulating peptide panel and identified proteins could serve as potential biomarkers for the early detection and diagnosis of ESCC. Nevertheless, a larger cohort will be required for further unequivocal validation of their clinical application.

## INTRODUCTION

Esophageal cancer is the eighth most common cancer and the sixth leading cause of cancer-related death worldwide [1]. Esophageal squamous cell carcinoma (ESCC) accounts for nearly 90% of esophageal cancer cases in Asian countries [2]. The average 5-year survival rate of ESCC is 30–40%, despite survival among early stage ESCC patients exceeding 90% [2, 3]. Unfortunately, due to the lack of early symptoms and reliable diagnostic techniques, over 70% of ESCC cases are diagnosed in advanced stages [2, 4]. Importantly, there are no established serological tumor markers for ESCC. It was reported that squamous cell carcinoma antigen (SCC-Ag) showed only 8–37% sensitivity for ESCC, while cytokeratin 19 fragment (Cyfra) 21–1 displayed some diagnostic value; the sensitivity and specificity of this latter marker reached 32–45% and 97.3%, respectively [5–7]. However, the combination of SCC-Ag and Cyfra 21–1 displayed a sensitivity of approximately 50% [5, 7].

The peptidome refers to the low-molecular-weight proteome of serum protein fragments and peptides and represents an emerging tool for biomarker discovery [8, 9]. The breakdown of large proteins often involves proteolytic processing. The progression of malignancy is accompanied by alterations in protease activities, thus affecting the constitution of endogenous peptides that are eventually secreted or diffused into the bloodstream. Indeed, in recent years, mass spectrometry-based serum peptide screening has been developed and applied as a high-throughput approach to discover potential diagnostic and prognostic biomarkers for various diseases [8, 9]. However, serum peptidome studies on ESCC remain very limited. For instance, Xu et al. explored the serum peptide fingerprints of ESCC in 139 patients and 49 controls using surface-enhanced laser desorption/ionization-time of flight-mass spectrometry (SELDI-TOF-MS) technology, and a six-peak diagnostic pattern was generated to achieve 97.1% sensitivity and 83.8% specificity [10]. However, the shortcoming of SELDI-TOF-MS is its low resolution and reproducibility. In addition, the peptides of interest can be difficult to identify. Recently, magnetic bead-based matrix-assisted laser desorption/ionization time-of-flight mass spectrometry (MB-based MALDI-TOF-MS) technology was also used for ESCC. Liu et al. established a peptide pattern with approximately 90% sensitivity and specificity in 38 healthy controls and 62 patients with ESCC [11]. In addition, Fan et al. built a diagnostic model of ESCC with almost 100% accuracy in 31 healthy volunteers and 32 ESCC patients [12], and Wan et al. reported an 11-peak pattern to distinguish ESCC, chemotherapy and tumor metastasis from healthy individuals with 100% correct prediction in 61 ESCC patients and 20 healthy individuals [13]. However, these studies were preliminary with small sample sizes and lacked independent validation and peptide identification.

In the present study, we used weak cation exchange magnetic beads (WCX-MB) coupled with MALDI-TOF-MS to analyze proteotypic peptide profiles in complex serum samples collected from 477 cancer and healthy individuals (201 ESCC patients; 196 healthy controls; 80 other kinds of digestive tumor patients). A diagnostic model consisting of three differentially expressed peptides was established using a K-Nearest Neighbor (KNN) algorithm. After independent validation, the diagnostic performance of the model was compared with that of SCC-Ag and Cyfra 21–1. Moreover, the three diagnostic peptides were successfully identified using linear ion trap-Orbitrap-tandem mass spectrometry (LTQ-Orbitrap-MS/MS) and were further verified using immunohistochemical staining and enzyme-linked immunosorbent assays (ELISAs) in large sample sets. Our results suggest that the high-throughput magnetic bead-based MALDI-TOF-MS assay is capable of performing rapid proteotypic peptide analyses in serum samples, and the identified proteins and their derivative peptides will deepen our understanding of tumorigenesis and serve as potential serological biomarkers for ESCC.

## RESULTS

### Discovery screening of peptide differences between ESCC and healthy controls

To assess the precision and reproducibility of our proteomic data, sera from ten patients with ESCC were pooled, and the same MALDI-TOF-MS instrument was then used to run six within-run assays and six between-run assays to determine the deviation. The mean coefficient of variation (CV) for the within-run assays was 15.02% (12.0–20.9%) and was 16.7% (10.8–22.9%) for the between-run assays (Supplementary Figure S1). The CV value of the relative intensity of each peak was less than 30%, suggesting that our serum peptide profiling system had good repeatability.

**Figure 1:**
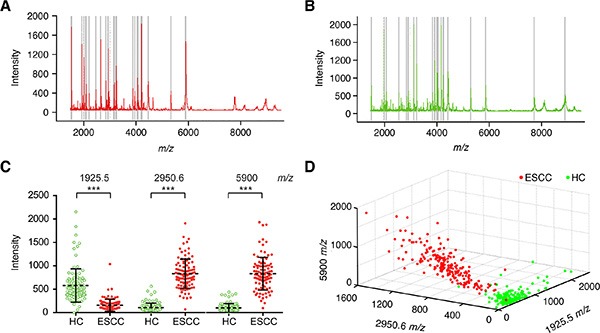
Serum peptide fingerprints in the training set Proteotypic peptides profilings for (**A**) ESCC (red, *n =* 100) and (**B**) healthy controls (green, *n =* 98) created by ClinTOF. x-axis, molecular mass (*m/z*); y-axis, relative intensity. The *m/z* ratio-intensity map (**C**) and three-dimensional pattern (**D**) of peaks at 1,925.5, 2,950.6 and 5,900.0 *m/z*.

In the discovery phase, 100 ESCC patients and 98 control samples were compared. The peak number and intensity of the serum peptide profiles between these two groups were completely different (Figure 1A, 1B). A total of 95 informative peaks with detection rates in all samples higher than 80% were detected by *m/z* spectra ranging from 1,000 to 10,000 Da. Twenty-one out of 95 features were significantly different between the ESCC patients and healthy controls, with a false discovery rate (FDR)-adjusted *P* < 0.05 and an average intensity higher than 300. Four mass peaks were down-regulated, whereas the other seventeen peaks were up-regulated, in the ESCC group (Table 1).

**Table 1:** The significantly differentially expressed mass peaks and identified peptides

Mean*m/z*	FDR-adjusted *P value*	Control		ESCC		Tendency	Identified peptide sequence	Identified proteins
		Mean	SD	Mean	SD			
5,924.6	< 1e-6	50.61	42.26	425.63	137.32	↑	Unknown peptide	Unknown peptide
5,910	< 1e-6	87.39	82.13	739.57	321.32	↑	Unknown peptide	Unknown peptide
2,950.6	< 1e-6	84.95	78.50	666.08	248.83	↑	T.NRIPESGGDNSVFDIFELTGAARKGSGR.R	Thrombospondin-1, TSP1
5,900	< 1e-6	100.55	92.86	831.12	350.57	↑	K.SSSYSKQFTSSTSYNRGDSTFESKSYKMADEAGSEADHEGTHSTKRGHAKSRPV.R	Fibrinogen A alpha-chain, FGA
5,882.1	< 1e-6	114.65	135.18	681.97	302.45	↑	Unknown peptide	Unknown peptide
1,925.5	< 1e-6	590.32	355.75	149.51	128.41	↓	F.MGVVSLGSPSGEVSHPRKT.R	Alpha2-HS glycoprotein, AHSG
4,209.6	< 1e-6	696.17	317.46	1772.59	547.33	↑	Unknown peptide	Unknown peptide
2,102.7	< 1e-6	266.24	91.47	532.28	130.13	↑	S.SSYSKQFTSSTSYNRGDST.F	Fibrinogen A alpha-chain, FGA
3,273	< 1e-6	764.46	443.45	221.86	149.88	↓	R.MNFRPGVLSSRQLGLPGPPDVPDHAAYHPF.R	Inter-alpha (globulin) inhibitor H4 (plasma Kallikrein-sensitive glycoprotein), ITIH4
4,199.6	< 1e-6	867.09	377.46	1767.14	553.09	↑	Unknown peptide	Unknown peptide
3,883.6	< 1e-6	241.78	123.66	489.36	167.81	↑	Unknown peptide	Unknown peptide
4,225.6	< 1e-6	224.98	173.97	526.85	219.50	↑	Unknown peptide	Unknown peptide
3,239.9	< 1e-6	325.76	185.87	615.66	190.27	↑	K.SYKMADEAGSEADHEGTHSTKRGHAKSRPV.R	Fibrinogen A alpha-chain, FGA
1,935.9	< 1e-6	2,456.36	1,104.97	1,018.23	623.57	↓	F.LTKKFSRHHGPTITAKL.Y	Complex-forming glycoprotein HC (alpha 1-microglobulin), AMBP
3,249.2	< 1e-6	269.90	169.20	482.98	150.94	?↑	Unknown peptide	Unknown peptide
4,086.1	< 1e-6	280.56	90.84	428.36	117.68	↑	Unknown peptide	Unknown peptide
2,669.1	< 1e-6	190.00	106.32	417.72	299.95	↑	R.NVHSGSTFFKYYLQGAKIPKPEAS.F	Inter-alpha (globulin) inhibitor H4 (plasma Kallikrein-sensitive glycoprotein), ITIH4
2,026.8	< 1e-6	1,290.54	1,002.81	2,730.99	1,205.03	↑	R.QLGLPGPPDVPDHAAYHPF.R	Inter-alpha (globulin) inhibitor H4 (plasma Kallikrein-sensitive glycoprotein), ITIH4
3,264.9	< 1e-6	880.07	424.43	470.60	201.01	↓	Unknown peptide	Unknown peptide
3,875.2	< 1e-6	298.70	136.42	489.05	168.85	↑	Unknown peptide	Unknown peptide
1,532.1	< 1e-6	527.84	317.26	1,022.00	507.38	↑	S.CSRDNTLKVIDLR.V	ATG16 autophagy related 16-like 2 (S. cerevisiae), isoform CRA_b

### Establishment of an ESCC diagnostic model and independent blind validations

The distinguishing ability of 21 differentially expressed peaks was firstly evaluated by receiver operating characteristic (ROC) curve analysis (Supplementary Figure S2). Eight out of 21 peaks showed outstanding classifier performance with the area under the curve (AUC) higher than 0.95 (Supplementary Figure S2, Supplementary Table S1). Furthermore, 5 peaks with an average peak intensity higher than 500 in either the ESCC or healthy control group as well as a minimum 4-fold change between the ESCC and healthy control groups were kept (1,925.5 *m/z*, 2,950.6 *m/z*, 5,900 *m/z*, 5,882.1 *m/z* and 5,910 *m/z*). The mean *m/z* values of peaks at 5,900 *m/z*, 5,882.1 *m/z* and 5,910 *m/z* were very close. The middle peak at 5,900 *m/z* was selected in combination with the 1,925.5 *m/z* and 2,950.6 *m/z* peaks. In addition, the KNN algorithm was utilized to generate a discriminatory model that distinguished ESCC patients from healthy controls in the training set (Supplementary Table S2). Finally, the 1,925.5 *m/z*, 2,950.6 *m/z* and 5,900.0 *m/z* pattern displayed very comparable diagnostic accuracy but with smaller indices and was thus used as a class predictor (Supplementary Table S2, Figure 1C, 1D). The peptide with a molecular weight of 1,925.5 Da was down-regulated in the ESCC group, while the peptides with molecular weights of 2,950.6 Da and 5,900 Da were up-regulated. In the training set, the sensitivity and specificity of our ESCC diagnostic model were 97.00% (97/100) and 95.92% (94/98), respectively.

To verify the accuracy of our identified classification model with the selected peptides, we introduced two independent cohorts as validation sets. The first cohort consisted of 101 ESCC patients and 98 healthy controls; its sensitivity and specificity were 97.03% (98/101) and 100.00% (98/98), respectively. The total accuracy was 96.46% (191/198) in the training set and 98.49% (196/199) in validation set 1. The second cohort included 80 cases with other kinds of digestive cancers, including 27 liver, 28 gastric and 25 colorectal cancer patients. The KNN model of ESCC classified 52 of 80 samples as positive and the other 28 as negative in this set. These results indicate that our diagnostic model is relatively specific for ESCC.

**Figure 2:**
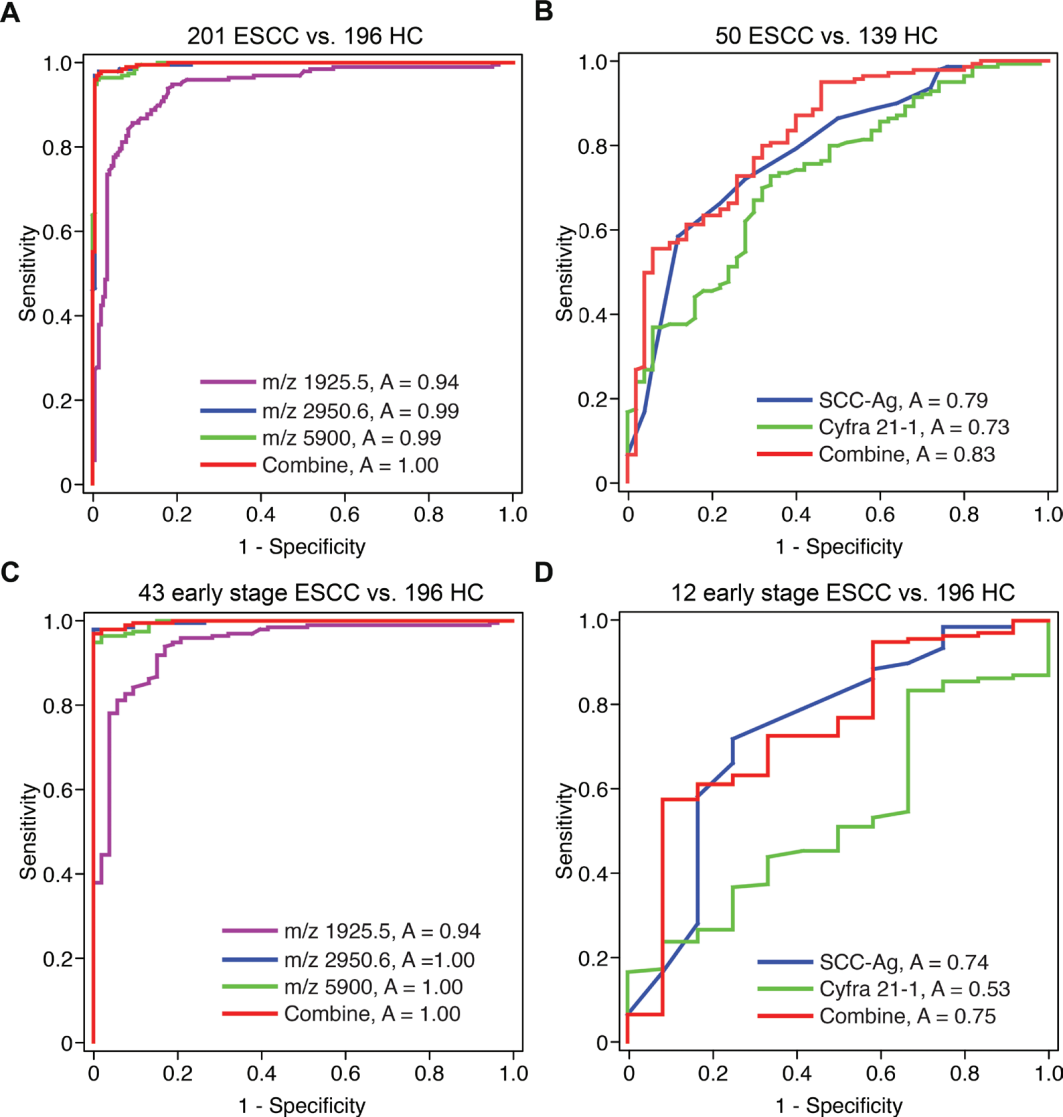
The ROC curves of the ESCC serum peptide diagnostic model The ROC curves of (**A**) the serum peptide diagnostic model (ESCC, *n* = 201; healthy controls, *n* = 196), (**B**) SCC-Ag and Cyfra 21–1 (ESCC, *n* = 50; healthy control, *n* = 139), (**C**) the serum peptide diagnostic model for early stage ESCC and (**D**) SCC-Ag and Cyfra 21–1 for early stage ESCC.

The diagnostic capability of each peak was further determined by the ROC curve. As shown in Figure 2A, the AUCs of three peptide peaks in the diagnostic model were 0.94 (95% confidence interval [CI]: 0.91–0.96), 0.99 (95% CI: 0.99–1.00) and 0.99 (95% CI: 0.99–1.00). The AUC of the whole model was 0.99 (95% CI: 0.99–1.00), with a sensitivity and specificity of 97.45% (95% CI: 94.15–99.17%) and 98.51% (95% CI: 95.70–99.69%), respectively.

### A comparison of the serum Cyfra 21–1 and SCC-Ag levels with the diagnostic model

Serum Cyfra 21–1 and SCC-Ag were also measured in 50 ESCC patients and 139 healthy controls. The Cyfra 21–1 levels were significantly higher in the ESCC patients (median, 3.43 ng/mL) than in the healthy controls (median, 2.18 ng/mL) (Mann-Whitney test, *P* < 0.001), and serum SCC-Ag was also elevated in the ESCC patients (median, 1.54 ng/mL) compared with the healthy controls (median, 0.77 ng/mL) (Mann-Whitney test, *P* < 0.001).

The specificity and sensitivity of serum Cyfra 21–1 and SCC-Ag were evaluated using ROC curves. The AUCs were 0.73 (95% CI: 0.65–0.81) for Cyfra 21–1, 0.79 (95% CI: 0.71–0.86) for SCC-Ag and 0.83 (95% CI: 0.76–0.89) for a combination of SCC-Ag and Cyfra 21–1 (Figure 2B). These results demonstrate that our ESCC serum peptides classifier model showed a significantly superior diagnostic capability compared with Cyfra 21–1 and SCC-Ag.

### The diagnostic model showed high performance for early stage ESCC

Furthermore, we compared the discriminative capability of our serum peptides classifier model on ESCC patients with different TNM stages. For stage 1, stage 2 and stages 3–4, the sensitivity in the training set and validation set 1 were 95.83% (23/24) and 100.00% (19/19), 93.55% (29/31) and 88.46% (23/26), and 100.00% (42/42) and 100.00% (49/49), respectively. ROC curve analysis also showed that for early stage ESCC (TNM stage 1), the AUCs of the three diagnostic peaks were 0.94 (95% CI: 0.91–0.96), 1.00 (95% CI: 0.99–1.00) and 1.00 (95% CI: 0.99–1.00) (Figure 2C). The whole model achieved a sensitivity of 96.94% (95% CI: 93.46–98.87%) and a specificity of 100.00% (95% CI: 93.28–100.00%) for early stage ESCC.

In comparison, the AUCs of SCC-Ag, Cyfra 21–1 and the combination of SCC-Ag and Cyfra 21–1 for the detection of early stage ESCC were 0.74 (95% CI: 0.57–0.91), 0.53 (95% CI: 0.38–0.69) and 0.75 (95% CI: 0.59–0.90), respectively (Figure 2D). The sensitivity and specificity of SCC-Ag and Cyfra 21–1 were only 57.55% (95% CI: 48.89–65.89%) and 91.67% (95% CI: 61.52–99.79%), respectively. These results indicated that our classifier model showed superior diagnostic value in early stages of ESCC.

### The identification of peptide peaks for ESCC

**Figure 3:**
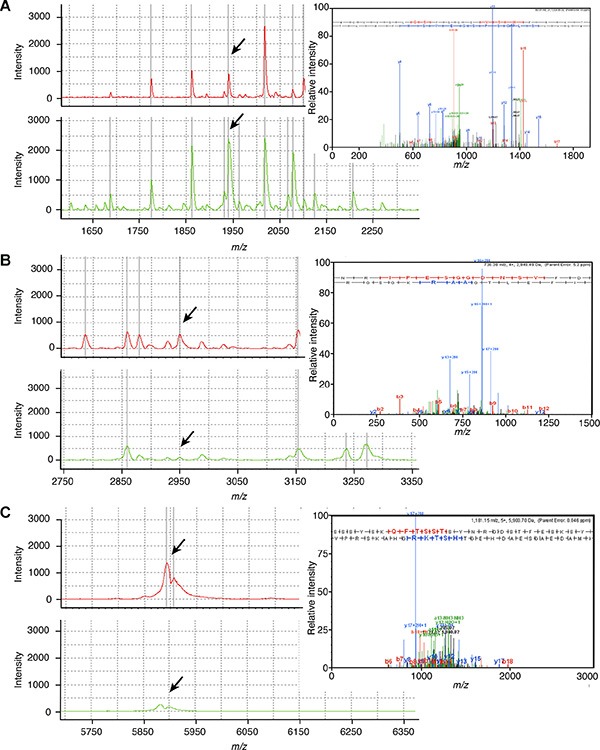
The identification of ESCC serum peptides The purified peptides from representative ESCC patients (red line) and healthy controls (green line) using magnetic beads were sequenced by LTQ-Orbitrap-MS/MS. The fragment ion spectra shown in the upper-right corner of (**A**) AHSG (sequence: F.MGVVSLGSPSGEVSHPRKT.R; 1925.5 Da), (**B**) TSP1 (sequence: T.NRIPESGGDNSVFDIFELTGAARKGSGR.R; 2950.6 Da) and (**C**) FGA (sequence: K.SSSYSKQFTSSTSYNRGDSTFESKSYKMADEAGSEADHEGTHSTKRGHAKSRPV.R; 5900 Da) were taken for a MS/MS ion search of IPI. The b and y fragment ion series are indicated together.

The peptides purified by magnetic beads were sequenced using LTQ-Orbitrap-MS/MS. Ten out of 21 peptides with differential expression between the ESCC and healthy control groups were successfully identified (Table 1). Among them, MS/MS analysis of the down-regulated peak at 1,925.5 Da revealed the sequence as F.MGVVSLGSPSGEVSHPRKT.R, which corresponded to alpha-2-HS-glycoprotein (AHSG) (Figure 3A). Moreover, the two up-regulated peaks at 2,950.6 Da and 5,900.0 Da were sequenced as T.NRIPESGGDNSVFDIFELTGAARKGSGR.R and K.SSSYSKQFTSSTSYNRGDSTFESKSYKMADEAGSEADHEGTHSTKRGHAKSRPV.R, which are unique to thrombospondin-1 (TSP1) and the fibrinogen alpha (FGA) chain, respectively (Figure 3B, 3C).

### TSP1 was overexpressed and associated with survival in human ESCC samples

**Figure 4:**
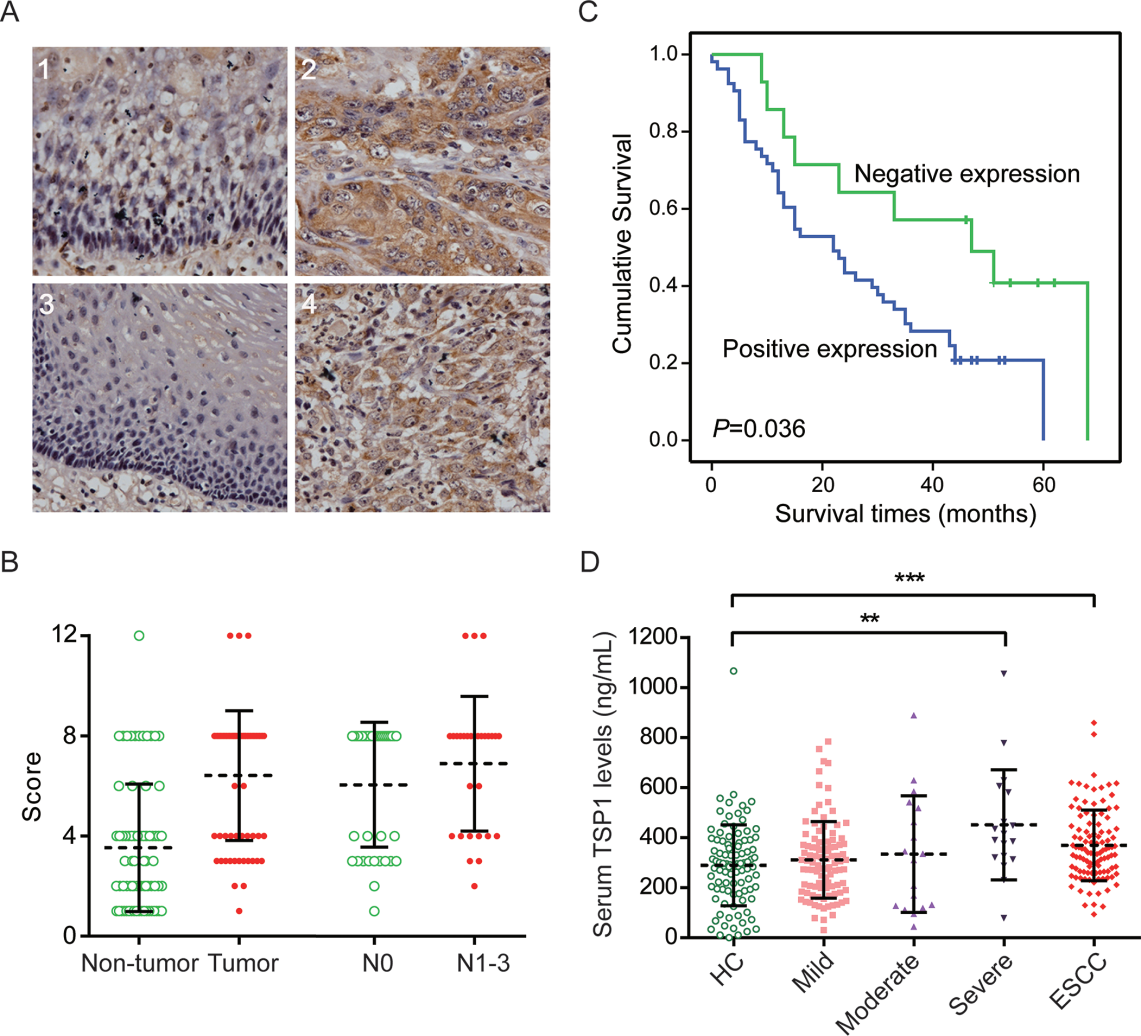
TSP1 was overexpressed in ESCC patients (**A**) Representative immunohistological staining of TSP1 in non-tumorous esophageal mucosa (1 and 3) and ESCC tissues (2 and 4), × 200. TSP1 was significantly differentially expressed and correlated with positive lymph node metastasis (**B**) and poor prognosis (**C**). (**D**) The distribution of serum TSP1 levels among the controls and the pre-cancerous dysplasia and ESCC patients.

The fragment of TSP1 was found to be up-regulated in the sera of ESCC patients. To explore whether this reflected the abnormal expression of TSP1 in ESCC tissues, an immunohistochemical staining assay was carried out on tissue microarrays. The results showed that TSP1 was mainly localized in the cytoplasm (Figure 4A) and was positively expressed in 78.5% (51/65) of tumor tissues but in only 45.3% (34/75) of non-cancerous tissues. The overexpression of TSP1 in ESCC tumor tissues was statistically significant (Chi-square test, *P* < 0.001, Supplementary Table S3). Meanwhile, TSP1 expression was stronger in patients with positive lymphatic metastasis (Fisher's exact test, *P =* 0.049, Figure 4B).

Notably, Kaplan-Meier survival analysis with the log-rank test showed a correlation between positive TSP1 expression and a shorter overall survival time (Log-rank test, *P =* 0.036) in ESCC patients (Figure 4C). The median survival times of the positive and negative expression groups were 22 and 47 months, respectively. This finding was further confirmed by univariate and multivariate Cox regression analyses (Supplementary Table S4). In the univariate analysis, patients with positive TSP1 expression exhibited a 2.21-fold increase in the relative risk (RR) for death (*P =* 0.043). Other significant risk factors included tumor size (*P =* 0.037), the depth of the tumors (*P =* 0.020), lymph node metastasis (*P =* 0.041) and TNM staging (*P =* 0.002). As concluded by the multivariate analysis, only positive TSP1 expression (RR = 3.00, *P =* 0.010) and the depth of the tumors (RR = 3.18, *P =* 0.023) were independent prognostic factors. Thus, positive expression of TSP1 was an independent prognostic factor in ESCC patients.

### TSP1 serum levels were higher in ESCC patients

To assess the serum TSP1 levels and their association with ESCC progression, we further measured TSP1 in the sera of healthy controls (*n =* 107) and patients with esophageal mild (*n =* 100), moderate (*n =* 18) and severe (*n =* 19) dysplasia and ESCC (*n =* 112) with ELISA. Compared with the healthy controls (median, 299.83 ng/mL), the TSP1 levels were significantly higher in the ESCC patients (median, 358.89 ng/mL, Mann-Whitney test, *P* < 0.001) and individuals with severe dysplasia (median, 390.52 ng/mL, Mann-Whitney test, *P =* 0.002, Figure 4D). Importantly, an increasing trend was observed during the precancerous progression of ESCC, from mild (median, 299.40 ng/mL) to moderate (median, 323.03 ng/mL) and severe dysplasia; this finding was not age- or gender-dependent in the healthy donors (Spearman's rank correlation test for age, *P =* 0.314; Mann-Whitney test for gender, *P =* 0.617). Additionally, there was no correlation between TSP1 expression and other histopathological features in our samples (Supplementary Table S5).

## DISCUSSION

In the present study, we utilized WCX-MB coupled with MALDI-TOF-MS to analyze 477 serum samples. We established a genetic algorithm model to diagnose ESCC with three significant peaks. The sensitivity and specificity of the model were over 96% in both the training and validation sets, and these values were significantly better than those of other well-studied serum proteins such as SCC-Ag and Cyfra 21–1. Importantly, we also demonstrated superior diagnostic performance for early stage ESCC patients with a sensitivity of 96.94%. Furthermore, the three diagnostic peptides were identified by LTQ-Orbitrap-MS/MS. The TSP1 protein was confirmed to be overexpressed in ESCC tumor tissues and sera specimens. Moreover, tissue TSP1 was an independent unfavorable prognostic factor, suggesting that its serum peptide level may represent a surrogate of tumor initiation and the progression of ESCC. To the best of our knowledge, this is the largest scale and most comprehensive study of serum peptide profiling in ESCC patients.

AHSG is a 63-kDa serum glycoprotein mainly synthesized by the liver. Previous studies have suggested that AHSG stimulates several processes, such as brain development, bone remodeling, the inhibition of insulin receptors, TGF-β-mediated signaling and breast cancer tumorigenesis [14–16]. Notably, human AHSG protein consists of two chains: the heavy chain A comprises residues 19–300, and the light chain B contains residues 341–367, whereas the middle residues 301–340 are considered to be a connecting peptide. Our identified fragment sequence (residues 321–339) is located in the connecting peptide region. Chymotrypsin can attack this fragment at position 320/321, and exopeptidase can release the terminal residue of Arg340 [17], thus leading to the formation of our identified fragment. Our results revealed that the peak intensity of 1925.5 *m/z* was decreased three-fold in the sera of ESCC patients. Furthermore, our immunohistochemical staining confirmed that AHSG was negatively expressed in ESCC tumor and non-tumor tissues (data not shown), indicating that the alteration of this fragment might reflect a hypothetical reduction in protease activity in ESCC patients.

The other diagnostic peak, 2,950.6 *m/z*, was identified as residues 19–46 of TSP1. The intensity of this peptide was increased 8-fold in ESCC patients compared with healthy controls. TSP1 can be secreted by multiple types of blood cells, fibroblasts, endothelial cells and even tumor cells [18]. Numerous investigators support the opinion that TSP1 is a multi-functional protein, and its biological activities and pathological roles in malignancy are complex and controversial [18]. In some cases, TSP1 possesses an angioinhibitory effect, resulting in the inhibition of tumor growth and experimental metastasis [19]. Indeed, reduced levels of TSP1 have been observed in bladder, lung and pancreatic cancer [20–22]. In contrast, TSP1 is overexpressed in the metastatic lesions of colon tumors, uterine leiomyosarcoma and breast cancer [23–25]. One possible mechanism to explain this phenomenon is that there are two temporally distinct phases to the effect of TSP1 on cancer progression. During the early stage, TSP1 inhibits neovascularization and prevents tumor growth. At a later stage, TSP1 may function as an adhesive protein or a modulator of extracellular proteases to promote tumor invasion [23]. Therefore, TSP1 in tumor tissues plays different roles depending on the tissue and cell context. In ESCC, TSP1 is overexpressed and correlated with regional lymph node invasion [26]. Our results showed that the N-terminal peptide derived from TSP1 and the total protein were significantly elevated in the tissue and serum of ESCC patients, reflecting the tumorigenesis and progression of ESCC, indicating that both of these indicators may serve as potential markers of ESCC.

The third peak in our model, at 5,900.0 *m/z*, was identified as FGA and consists of one of three polypeptide chains of fibrinogen. As a major serum high-abundance protein secreted by hepatocytes, the fragments of FGA have been identified as decreased or increased in several serum/plasma peptide profiling studies [27–29]. Our identified peptide sequence mapped to the C-terminal end of residues 576–629. In addition, this sequence was longer than those described in previous reports, and its intensity was increased by more than 8-fold in ESCC patients. Our immunohistochemical staining revealed that there were no significant differences in the expression of FGA between tumor and non-tumor tissue samples from ESCC patients (49.15% vs. 58.33%, Chi-square test, *P =* 0.294). Therefore, the overexpression of this FGA peptide (5,900 Da) mainly reflects the increased protease activities in ESCC tissues.

Fibrinogen plays a central role in coagulation. Degraded fibrinogen fragments also possess biological functions, including vasoactive effects, mitogenic effects and migratory effects [29]. Additionally, hyperfibrinogenemia reflects a state of hypercoagulation and thrombocytosis and is related to malignant growth [30] and hematogenous metastasis [31]. Indeed, plenty of studies have reported that elevated fibrinogen levels are associated with poor prognoses of various tumors, including ESCC [30, 32]. In addition, it has been reported that the N-terminal sequence of TSP-1 (aa 169–182) can bind to fibrinogen and inhibit osteosarcoma cell-induced platelet aggregation [33]. Thus, it seems likely that the 5,900.0 *m/z* peak reflected the status of hyperfibrinogenemia in ESCC patients. Meanwhile, the elevated levels of TSP-1 and FGA in ESCC patients might synergistically contribute to tumor progression and metastasis.

In addition to the three identified proteins, our study identified other peptides (Table 1). The differentiating patterns of ITIH4-derived peptides were previously reported in various cancer types [34]. Moreover, the aberrant expression of serum AMBP was previously identified in gastric cancer [35]. Using a strictly matched case-control study design, our diagnostic model and identified peptides were found to be significantly tumor-related. A larger cohort will be required for further unequivocal validation of their clinical application.

In conclusion, we constructed a diagnostic model consisting of three peptide peaks and achieved high sensitivity, specificity and over 97% accuracy to discriminate ESCC patients from healthy controls. Importantly, the diagnostic value of our model was outstanding for early stage ESCC with a sensitivity of 96.94% and a specificity of 100%, which was significantly superior to SCC-Ag and Cyfra 21–1. The high-throughput magnetic bead-based MALDI-TOF-MS assay is capable of performing rapid proteotypic peptide analyses in complex serum samples. One of the identified protein, TSP1, was significantly overexpressed in ESCC patients, and the fluctuation of serum TSP1 levels reflected disease progression. In addition, TSP1 was an independent poor prognostic factor in ESCC patients. Together, these findings suggest that circulating peptides may provide information on ESCC carcinogenesis and act as potential biomarkers for early diagnosis and prognosis prediction.

## MATERIALS AND METHODS

### Sample collection and preparation

We collected patient samples (Table 2) according to the Ethics Committee of the Cancer Hospital, Chinese Academy of Medical Sciences (Beijing, China) under approval # 12–130/664. A total of 201 patients with sporadic ESCC (male/female: 160/41; median age, 59 ± 9.4 SD; range 16–82 years) and 80 patients with other types of digestive cancer (27 liver, 28 gastric and 25 colorectal cancers; male/female: 59/21; median age, 54 ± 12.0 SD; range 31–77 years) were enrolled in this study from January 2009 to March 2010. All patients were pathologically diagnosed as having ESCC or other digestive cancers by two senior pathologists, and serum was collected prior to surgical operations or chemo/radiotherapy. Meanwhile, 196 serum samples from healthy individuals (male/female: 162/34; median age, 59 ± 8.2 SD; range 44–76 years) were obtained from a healthy public population cohort in the Navy General Hospital (Beijing, China) who undergo medical examination annually and all healthy information were collected up to 7 years. The enrollment criteria for control subjects were as follows: 1) the absence of benign or malignant tumors; 2) no family history of cancer; 3) a qualified physical examination, finding no dysfunction of vital organs; and 4) frequency-matched for age, gender and geographic region with the ESCC cases. The serum samples were prepared according to a standard protocol [36]. Samples with chyle blood or hemolysis and more than 2 freeze/thaw cycles were excluded from further analysis.

**Table 2:** Clinical features of the ESCC patients and healthy controls

Characteristic	Training set			Validation set 1			Overall
	ESCC	Control		ESCC	Control		
	*n* = 100 (50.5%)	*n* = 98 (49.5%)	*P* value^a^	*n* = 101 (50.8%)	*n* = 98 (49.2%)	*P* value	*P* value
Age (year)							
Median	60.39	59.15	0.309	59.99	60.02	0.981	0.496
Range	41–81	46–73		16–82	44–76		
Gender			0.221			0.916	0.517
Male	78	84		82	78		
Female	22	14		19	20		
TNM staging							
I	24			19			
II	31			26			
III	41			46			
IV	1			3			
Unknown	3			7			
Differentiation							
Well	15			29			
Moderately	50			52			
Poorly	30			10			
Unknown	5			10			

a Spearman's rank correlation test and the Chi-square test were used to compare the differences in age and sex between two groups, respectively.

All of the ESCC and healthy control samples were randomly divided into the training (100 ESCC patients and 98 healthy individuals) and validation (101 ESCC patients and 98 healthy individuals) sets. The 80 samples from non-ESCC tumor patients were used for additional validation. The ESCC tissue microarrays (TMAs) (Outdo Biotechnology, Shanghai, China) contained 65 primary tumor and 75 adjacent esophageal epithelia tissue samples.

### Serum pretreatment with magnetic beads, protein/peptide profiling and data processing

All of the serum samples were fractionated by WCX-MB according to the manufacturers’ instructions. Anchor chip spotting and protein/peptide profiling were performed as described previously [36]. For system quality control, 5 standard peptides were used as an external standard preparation to ensure the average molecular weight deviation was no more than 100 ppm. For data processing, all spectra obtained from the serum samples were analyzed using BioExplorer^™^ software (Bioyong Tech, Beijing, China). Each spectrum was normalized, baseline-corrected and smooth-applied using default parameters. The signal-to-noise (S/N) ratio was set to higher than five. To align the spectra, a mass shift of no more than 0.1% was determined. The peaks that were detected in more than 80% of samples were counted as informative peaks. The Wilcoxon test was used to compare the peak intensities in the two groups. Then, each statistic was corrected for multiple testing using the Benjamini method to control for the FDR. The peaks with adjusted *P* values < 0.05 and an average peak intensity higher than 300 were regarded as statistically significant. Thereafter, the KNN algorithm was used to establish the best pattern for distinguishing ESCC. After each profile was generated, a 20% leave out cross-validation process was performed.

### The identification of peptide biomarkers by LTQ-Orbitrap-MS/MS

The sequencing and identification of diagnostic peptides in the model were performed using a nano-LC/ESI–MS/MS system consisting of an Aquity UPLC system (Waters, MA) and a LTQ Orbitrap XL mass spectrometer (Thermo Fisher, MA) equipped with a nano-ESI source, as described previously [36]. The obtained chromatograms were analyzed with BioworksBrowser 3.3.1 SP1, and the resulting mass lists were used in a database search with Sequest™ (IPI Human (3.45)). Relative accuracy parameters for generating the peak list were set at 50 ppm and 1 Da for the parent ion and fragment mass, respectively. Positive protein identification was accepted for a peptide with Xcorr ≥ 3.75 for triply charged ions and 2.2 for doubly charged ions with ΔCn ≥ 0.1 and a peptide probability ≤ 1E-03.

### Serum Cyfra 21–1 and SCC-Ag measurement

Serum Cyfra 21–1 was analyzed using an electrochemiluminescence immunoassay (Elecsys@CYFRA21–1) in an automated Modular Analytics E170 analyzer (Roche, Germany). Serum concentrations of SCC-Ag were measured with a chemiluminescent microparticle immunoassay (CMIA) (Abbott, IL).

### Immunohistochemical staining

Immunohistochemical staining for TSP1, AHSG and FGA expression was performed on multi-tissue microarrays (MTAs) (Outdo Biotech, Shanghai, China). Tissues were stained with anti-TSP1 (Proteintech Group, IL), anti-AHSG (Sigma-Aldrich, MO) and anti-FGA (Abcam, UK) antibodies, and images were captured using Aperio ScanScope CS software (Vista). After calculating the informative cases, the intensity of staining in individual cases was quantified as previously described [37]. A score of 4–12 was defined as positive expression, and a score of 0–3 was considered negative.

### ELISA

The serum TSP1 levels were measured using a commercially available ELISA kit (Cloud-Clone Corp., TX) according to the manufacturer's instructions. The absorbance was measured at 450 nm using a Model 680 microplate reader (Bio-Rad Laboratory, CA).

### Statistical analysis

SPSS software v17.0 was used to calculate all statistical comparisons. Values of *P* < 0.05 were considered significant. ROC analyses were performed to calculate the AUCs to define the cutoff line for each serum peptide/protein. In this case, the logistic regression model was used to combine multiple biomarkers for diagnostic classification. The Kaplan-Meier method combined with log-rank analysis was performed to compare survival curves. Univariate and multivariate analyses were performed using the Cox regression model.

## SUPPLEMENTARY MATERIALS TABLES AND FIGURES


