# Supplementary material for: Novel circulating peptide biomarkers for esophageal squamous cell carcinoma revealed by a magnetic bead-based MALDI-TOFMS assay
Source: Oncotarget. 2016 Mar 16;7(17):23569–80. doi: 10.18632/oncotarget.8123 (PMC5029648; doi:10.18632/oncotarget.8123)
Supplement: Supplementary file 1 [file oncotarget-07-23569-s001.pdf]

Novel circulating peptide biomarkers for esophageal squamous cell carcinoma revealed by a magnetic bead-based MALDI-TOF-MS assay

Supplementary Materials

Supplementary Table S1: The comparison of the 21 significantly differentially expressed peaks

Mean *m/z*	Control		ESCC		ESCC/ Control ratio	AUC	Standard error	*P* value	95% CI	
	Mean	SD	Mean	SD					Lower	Upper
5924.6	50.61	42.26	425.63	137.32	8.41	1.00	0.0041	< 0.001	0.99	1.00
1925.5	590.32	355.75	149.51	128.41	0.25	0.95	0.0162	< 0.001	0.92	0.99
2950.6	84.95	78.50	666.08	248.83	7.84	0.99	0.0057	< 0.001	0.98	1.00
5900	100.55	92.86	831.12	350.57	8.27	0.99	0.0050	< 0.001	0.98	1.00
2102.7	266.24	91.47	532.28	130.13	2.00	0.95	0.0162	< 0.001	0.92	0.98
4209.6	696.17	317.46	1772.59	547.33	2.55	0.95	0.0145	< 0.001	0.92	0.98
5882.1	114.65	135.18	681.97	302.45	5.95	0.96	0.0121	< 0.001	0.94	0.99
5910	87.39	82.13	739.57	321.32	8.46	0.99	0.0041	< 0.001	0.98	1.00
1532.1	527.84	317.26	1022.00	507.38	1.94	0.78	0.0356	< 0.001	0.71	0.85
1935.9	2456.36	1104.97	1018.23	623.57	0.41	0.87	0.0263	< 0.001	0.82	0.92
2026.8	1290.54	1002.81	2730.99	1205.03	2.12	0.82	0.0300	< 0.001	0.76	0.88
2669.1	190.00	106.32	417.72	299.95	2.20	0.82	0.0287	< 0.001	0.77	0.88
3239.9	325.76	185.87	615.66	190.27	1.89	0.86	0.0262	< 0.001	0.81	0.91
3249.2	269.90	169.20	482.98	150.94	1.79	0.85	0.0289	< 0.001	0.79	0.90
3264.9	880.07	424.43	470.60	201.01	0.53	0.82	0.0309	< 0.001	0.76	0.89
3273	764.46	443.45	221.86	149.88	0.29	0.92	0.0199	< 0.001	0.88	0.96
3875.2	298.70	136.42	489.05	168.85	1.64	0.82	0.0301	< 0.001	0.76	0.87
3883.6	241.78	123.66	489.36	167.81	2.02	0.89	0.0227	< 0.001	0.85	0.94
4086.1	280.56	90.84	428.36	117.68	1.53	0.83	0.0302	< 0.001	0.77	0.89
4199.6	867.09	377.46	1767.14	553.09	2.04	0.90	0.0219	< 0.001	0.86	0.95
4225.6	224.98	173.97	526.85	219.50	2.34	0.87	0.0253	< 0.001	0.82	0.92

Supplementary Table S2: The diagnostic model based on different number of significantly differentially expressed peaks generated by KNN algorithm in the training set

	21 peaks	8 peaks	6 peaks	5 peaks	3 peaks
Sensitivity	98.00% (98/100)	97.00% (97/100)	97.00% (97/100)	97.00% (97/100)	97% (97/100)
Specificity	98.98% (97/98)	98.98% (97/98)	96.94% (95/98)	96.94% (95/98)	95.92% (94/98)
Accuracy	98.48% (195/198)	97.98% (194/198)	96.97% (192/198)	96.97% (192/198)	96.46% (191/198)

a, 1532.1, 1925.5, 1935.9, 2026.8, 2102.7, 2669.1, 2950.6, 3239.9, 3249.2, 3264.9, 3273.0, 3875.2, 3883.6, 4086.1, 4199.6, 4209.6, 4225.6, 5882.1, 5900.0, 5910.0, 5924.6 *m/z*.

b, 5924.6, 1925.5, 2950.6, 5900, 2102.7, 4209.6, 5882.1, 5910 *m/z*.

c, 5924.6, 1925.5, 2950.6, 5900, 5882.1, 5910 *m/z*.

d, 1925.5, 2950.6, 5900, 5882.1, 5910 *m/z*.

e, 1925.5, 2950.6, 5900.0 *m/z*.

Supplementary Table S3: Expression of TSP1 and its clinical significance in IHC

Variable	Total	TSP1 expression		*P* value[a]
		Positive, *N* (%)	Negative, *N* (%)	
Gender				0.205
Male	50	41 (80.4)	9 (64.3)	
Female	15	10 (19.6)	5 (35.7)	
Age				0.287
≥ 65	36	30 (58.8)	6 (42.9)	
< 65	29	21 (41.2)	8 (57.1)	
Pathological grade				0.139
I, I–II	25	22 (43.1)	3 (21.4)	
III, IV	40	29 (56.9)	11 (78.6)	
Tumor diameter				0.449
< 5	36	27 (52.9)	9 (64.3)	
≥ 5	29	24 (47.1)	5 (35.7)	
Depth of the tumors				0.451
T1 + T2	11	10 (15.9)	1 (7.1)	
T3 + T4	52	39 (84.1)	13 (92.9)	
Unknown	2	2	0	
Lymphatic metastasis				0.049
Positive (N1–3)	29	26 (51.0)	3 (21.4)	
Negative (N0)	36	25 (49.0)	11 (78.6)	

TNM staging				0.139
1B, 2	36	26 (54.2)	10 (76.9)	
3A, 3B, 4	25	22 (45.8)	3 (23.1)	
Unknown	4	3	1	
Tissue				< 0.001
Normal	75	34 (40.0)	41 (74.5)	
Cancer	65	51 (60.0)	14 (25.5)	

[a]Chi-square test or Fisher’s exact test.

Supplementary Table S4: Univariate and multivariate survival analysis for overall survival in ESCC

	Relative risk (95% CI)	*P* value
Univariate		
Age (> = 65 vs. < 65 years old)	1.15 (0.66–2.00)	0.623
Gender (female vs. male)	0.71 (0.34–1.45)	0.343
Pathological grade (II and III vs. I)	0.90 (0.51–1.60)	0.729
Tumor size (> = 5 vs. < 5 cm)	1.80 (1.04–3.13)	0.037
Depth of the tumors (T3 and T4 vs. T1 and T2)	3.02 (1.19–7.65)	0.020
Lymph node metastasis (N1–3 vs. N0)	1.78 (1.02–3.10)	0.041
TNM staging (3 and 4 vs. 1 and 2)	2.51 (1.39–4.12)	0.002
TSP1 expression (positive vs. negative)	2.21 (1.03–4.75)	0.043
Multivariate		
Depth of the tumors (T3 and T4 vs. T1 and T2)	3.18 (1.17–8.66)	0.023
TNM staging (3 and 4 vs. 1 and 2)	1.79 (0.96–3.35)	0.068
TSP1 expression (positive vs. negative)	3.00 (1.29–6.95)	0.010

Supplementary Table S5: Expression of serum TSP1 levels and their clinical significance

Variable	Total	TSP1 expression (ng/mL)		*P* value[a]
		Median	Range	
Age				0.236
< 65	70	364.5	129.9–859.7	
≥ 65	42	339.3	93.5–813.4	
Gender				0.713
Male	92	362.2	93.5–859.7	
Female	20	354.9	238.4–813.4	
Differentiation				0.262
Median, high	72	351.2	124.8–813.4	
low	38	408.3	93.5–859.7	
Unknown	2		322.0–423.1	
Tumor diameter (cm)				0.977
< 5	41	353.1	93.5–813.4	
≥ 5	67	366.3	124.8–859.7	
Unknown	4	-	263.2–502.9	
Lymphatic metastasis				0.335
Positive (N1–3)	56	354.1	93.5–859.7	
Negative (N0)	56	380.3	124.8–650.6	
TNM staging				0.388
I–II	54	358.9	93.5–859.7	
III–IV	56	361.5	129.9–650.6	
Unknown	2	-	186.1–381.8	

[a]Mann-Whitney test.

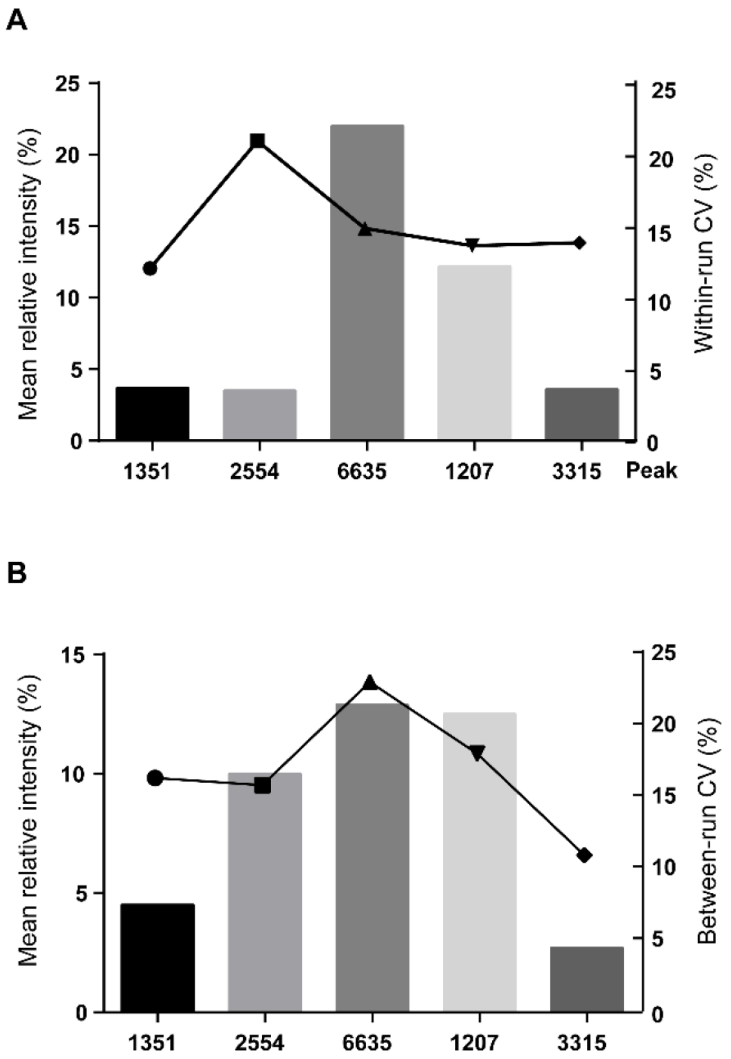

Supplementary Figure S1: Reproducibility of mass spectra profiled by magnetic beads and MALDI-TOF analysis. Five representative peaks of a pooled sera from 10 ESCC patients were used to estimate the variation of the MALDI-TOF instrument with six independent experiments. (**A**) The variation of within-run. (**B**) The variation of between-run.

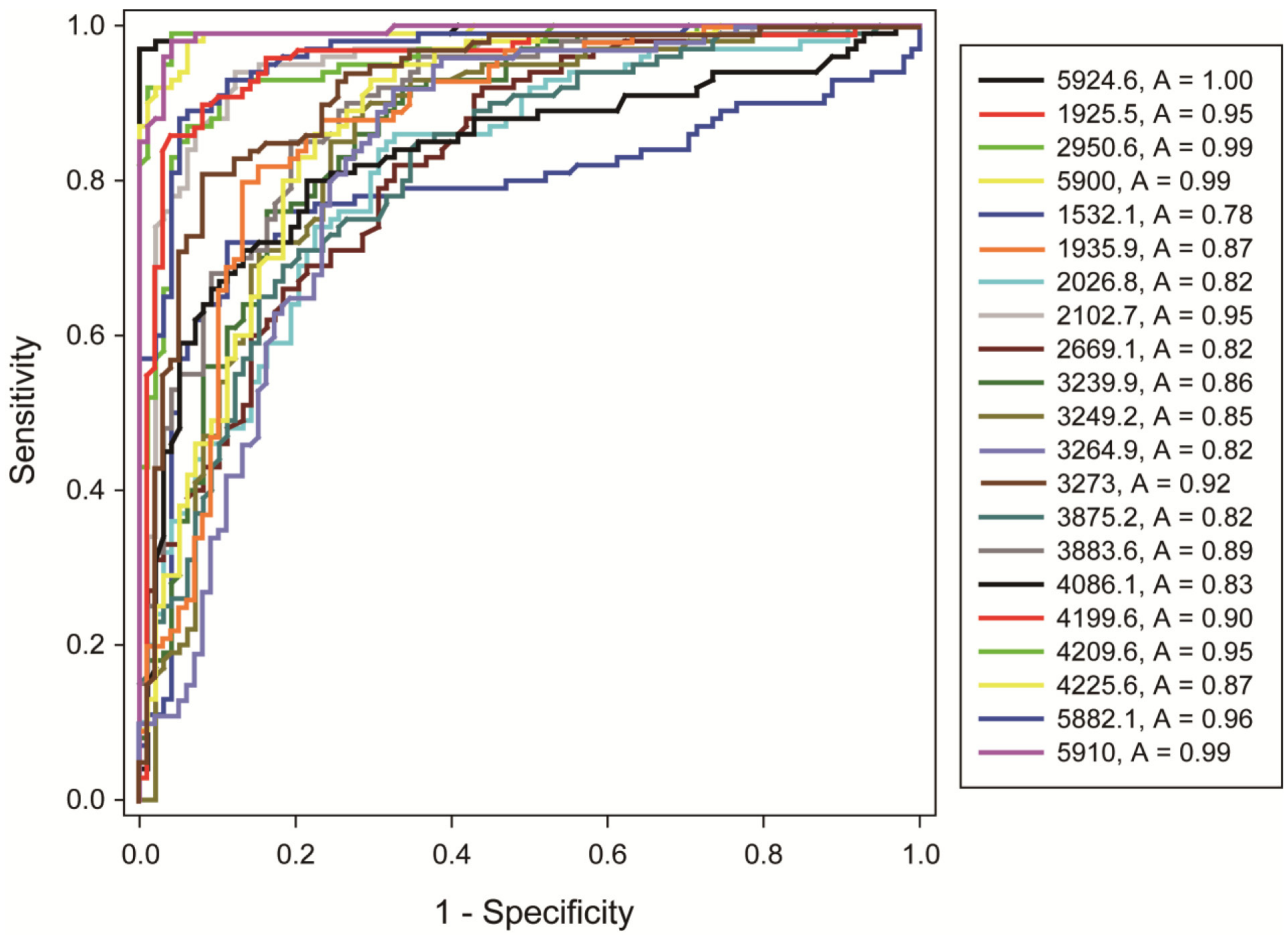

Supplementary Figure S2: The ROC curve of 21 differentially expressed peptide peaks in the samples of training set.
